# 

**Published:** 1887-08-13

**Authors:** 


					PRICE ONE PENNY.
No. 46, Vol. II. rssS^ W W
o
to August 13th, 1887.
*1 n Per Annum, 6s. 6d.,post free.
?    \1\ IRi j? f_er Annum, os. oqm post iree.
"l?red at the General Post Office
as a Newspaper.]
?t ik[ ?i Mr^l rer Annum> ea.,post rree.
\j^ Monthly Parts, 6d.; post free, 7id.
*-? / Ditto per Ann., 7s. 6d., post free.

				

